# Supplementary material for: All optical dynamic nanomanipulation with active colloidal tweezers
Source: Nat Commun. 2019 Sep 13;10:4191. doi: 10.1038/s41467-019-12217-2 (PMC6744401; doi:10.1038/s41467-019-12217-2)
Supplement: Supplementary file 1 — Supplementary Information [file 41467_2019_12217_MOESM1_ESM.pdf]

Supplementary information for:

All optical dynamic nanomanipulation with active colloidal tweezers

Ghosh et al

*Correspondence to: ambarish@iisc.ac.in

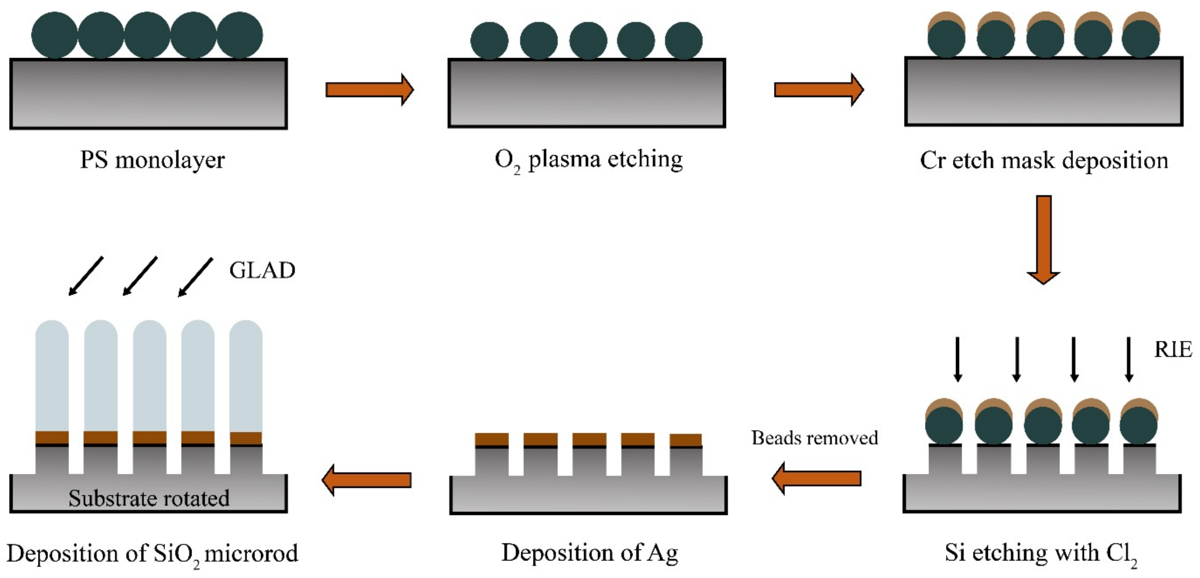

Supplementary Figure 1: Schematic description of ACT fabrication process.

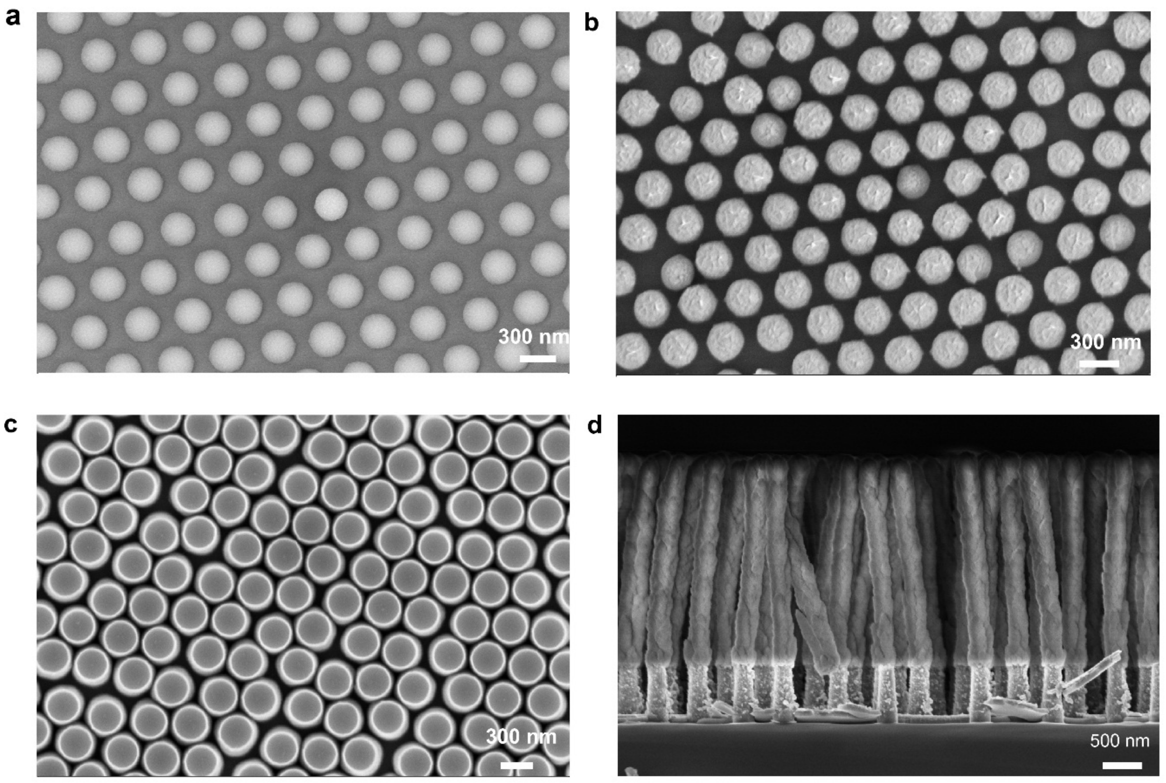

Supplementary Figure 2: SEM image at different steps of fabrication. (a) Monolayer of polystyrene beads on silicon substrate after oxygen-plasma etching. (b) Glancing angle deposition of Cr, only on the beads. (c) Reactive ion anisotropic etching of Si with Cl_2, forming nano-pillars with flat and nearly circular top (size distribution is plotted in Supplementary Figure 3a) across the entire wafer. (d) Growth of 50 nm Ag followed by 3 nm Ti and 2.5 μm SiO_2 on nano-pillars by glancing angle deposition.

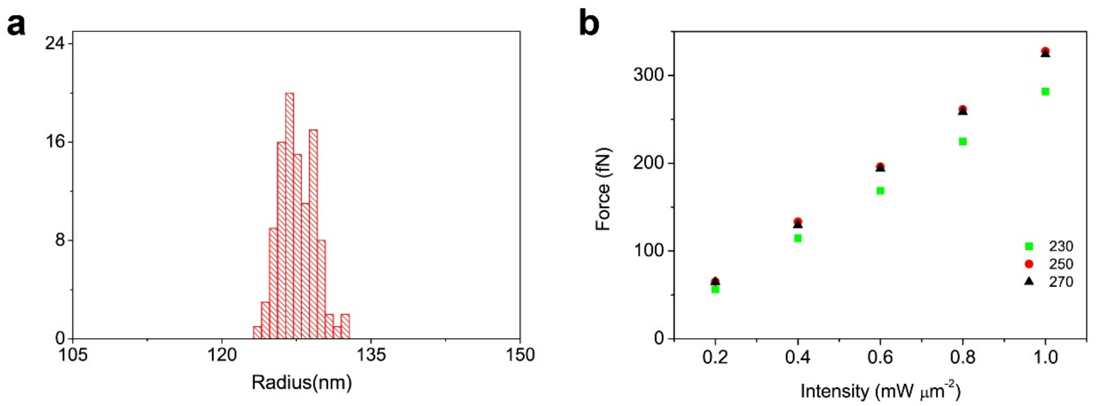

Supplementary Figure 3: Variation in nanopillar size and trapping force. (a) Size distribution of nanodisks. (b) Trapping force on 200 nm PS particles for 230, 250 and 270 nm size nanodisks. We assumed vertical configuration where the PS particles were placed in position I.

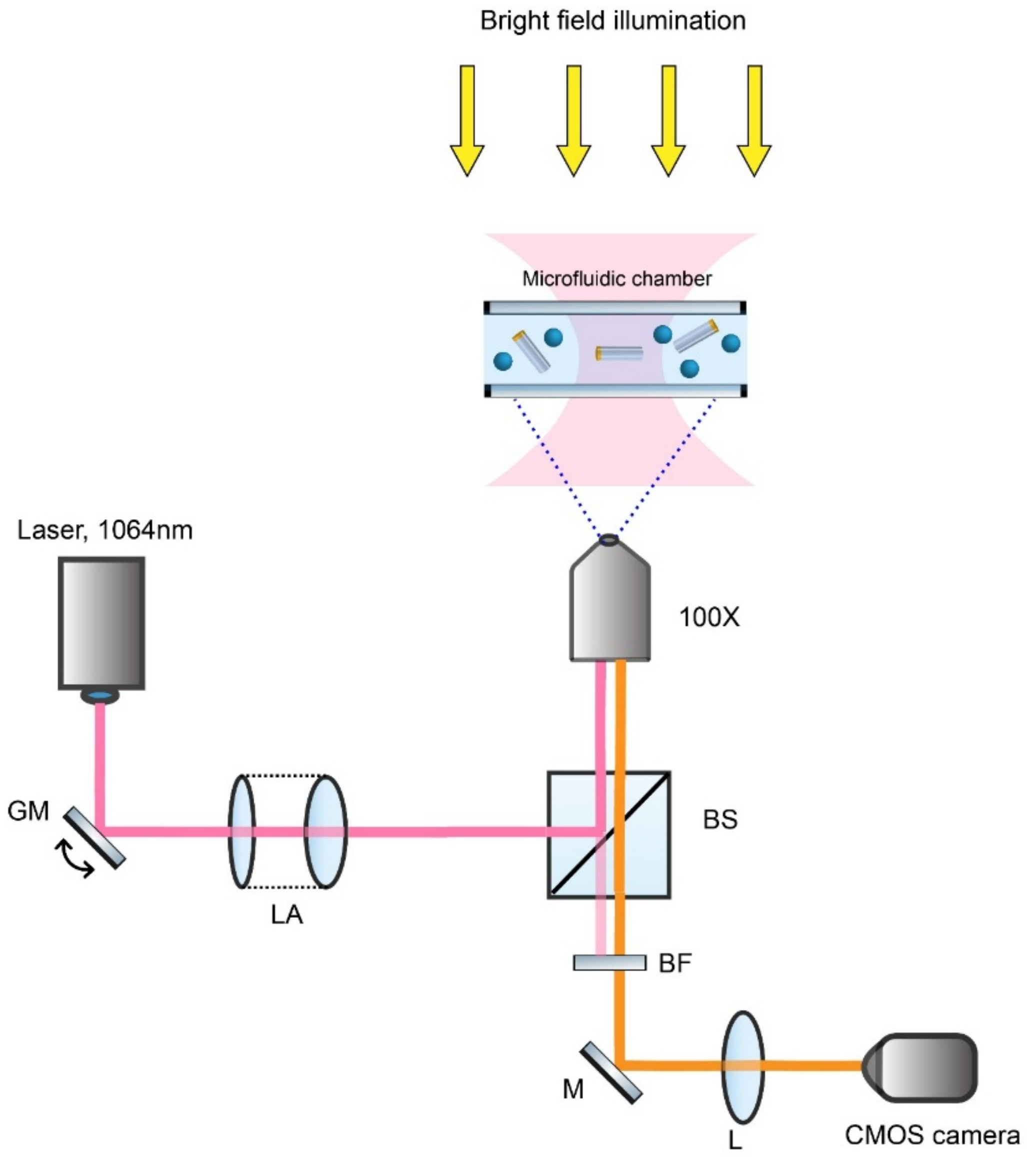

Supplementary Figure 4: Schematic of the experimental setup: Light from a 1064 nm Nd:YAG laser going through a telescopic lens assembly (LA) and beam splitter (BS) was transmitted through microscope objective illuminating a microfluidic chamber containing the ACTs and colloidal beads. The galvo-mirror (GM) was used to create multiple focused spots. The ACTs and the beads were imaged in a CMOS camera with bright field illumination from a Tungsten-Halogen lamp (marked in yellow) going through bandpass filter (BF), mirror (M) and lens (L).

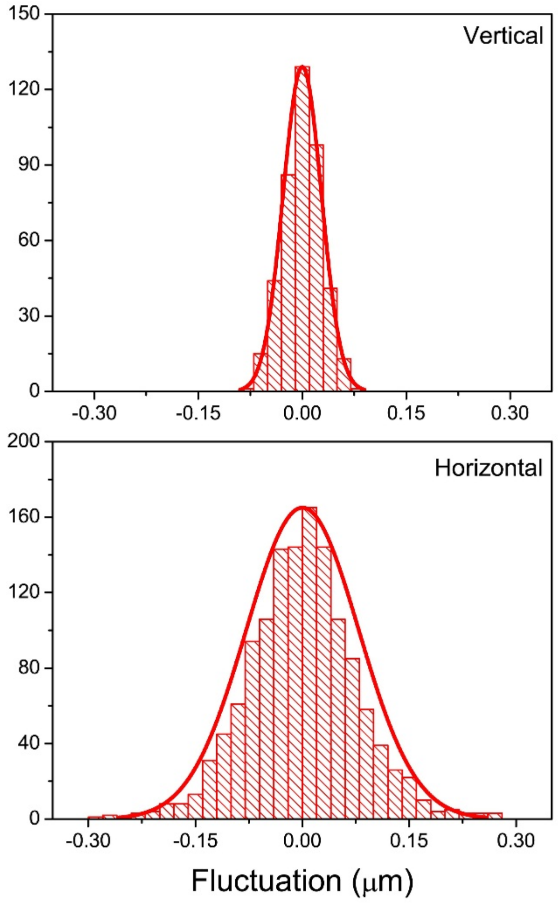

Supplementary Figure 5: Histogram of position fluctuations of ACTs at focused (vertical configuration) and defocused (horizontal configuration) laser.

Supplementary Note 1: Simulation of electromagnetic field and trapping force

We calculated the electric fields around the plasmonic nanodisk using finite element method in COMSOL Multiphysics, by solving the Helmholtz equation $\nabla \times (\nabla \times \boldsymbol{E}) - k_0^2 \varepsilon(\omega)\mathbf{E} = 0$. The relative permittivity values of silver were taken from experimental values[1] and for water we assumed $\varepsilon_m = 1.76$ to be constant at all wavelengths. The orientation of the nanodisk and polarization states of the incident beam have been mentioned in the main text. We used a spherical PML of thickness 200 nm and order one surrounding the model geometry. The PML was placed at $\frac{\lambda}{5}$ nm away from the disc. Each domain was meshed using free tetrahedral meshing of maximum size 42 nm outside the plasmonic disc and maximum element size of 15 nm inside the disc to obtain optimal convergence. We use direct solver to solve the wave equation and calculate the electric field distributions.

To obtain the total electromagnetic force exerted by the Ag nanodisk on the colloidal particle we have used Maxwell stress tensor ($\overleftrightarrow{\mathbf{T}}$) derived from the fields. The average mechanical force on the particles can be written as:

$$< \boldsymbol{F} >= \int < \overleftrightarrow{\mathbf{T}} >\cdot d\boldsymbol{S} \qquad (1)$$

and, $$\overleftrightarrow{\mathbf{T}} = \varepsilon_0 \varepsilon \boldsymbol{E}\boldsymbol{E} + \mu_0 \mu \boldsymbol{H}\boldsymbol{H} - \frac{1}{2}\left(\varepsilon_0 \varepsilon E^{\mathbf{2}} + \mu_0 \mu H^2\right)\overleftrightarrow{\mathbf{I}} \qquad (2)$$

where, ε and μ are electric permittivity and magnetic permeability of the surrounding medium respectively. The trapping potential ($U_{\text{trap}} = \frac{1}{2}\alpha\mathbf{E}^2$) for the trapped particle (Relative permittivity $\varepsilon = 2.56$) at the Rayleigh limit was obtained from

$$U_{\text{trap}} = \frac{3}{2}\varepsilon_0 \frac{\varepsilon - \varepsilon_m}{\varepsilon + 2\varepsilon_m} \int |\mathbf{E}|^2 dv \qquad (3)$$

Supplementary Note 2: Modelling of temperature rise

To find out temperature rise due to plasmon induced heating in Ag-nanodisks we need to know the absorbed thermal power density inside the nanostructures which can be written as,

$$Q(\boldsymbol{r}) = \frac{1}{2} Re[\mathbf{J}^*(\boldsymbol{r}) \cdot \mathbf{E}(\boldsymbol{r})] \qquad (4)$$

where $\boldsymbol{J}(\boldsymbol{r})$ is the complex amplitude of the electronic current density inside the Ag nanostructure, written as $\boldsymbol{J}(\boldsymbol{r}) = \mathrm{i}\boldsymbol{\omega}\mathbf{P}$ and $\mathbf{P}(\boldsymbol{r}) = \varepsilon(\omega)\mathbf{E}(\boldsymbol{r})$. The estimation of electric field $\boldsymbol{E}(\mathbf{r})$ was obtained by solving the time-independent Helmholtz equation for 250 nm Ag nanodisk of thickness 50 nm (details in previous section),

$$\nabla \times (\nabla \times \mathbf{E}) - k_0^2 \varepsilon(\omega)\mathbf{E} = 0 \qquad (5)$$

where light absorption in water and silica is ignored. The temperature distribution in ACT and its outside is governed by

$$\nabla \cdot [\kappa \nabla \mathrm{T}(\mathbf{r})] + \rho C_p \frac{dT}{dt} = Q(\mathbf{r}) \qquad (6)$$

where ρ is mass density (kg m^{-3}), κ is thermal conductivity ($Wm^{-1}K^{-1}$) and C_p is thermal heat capacity (J kg^{-1} K^{-1}). The resulting temperature can be assumed to be uniform over the metallic region due to large contrast of thermal diffusivity D in metal ($D \sim 10^{-4}$ m^2s^{-1}) and surrounding dielectric ($D \sim 10^{-7}$ m^2s^{-1}). As $\left(\frac{\kappa}{\rho C_p}\right)_{\text{silver}} >> \left(\frac{\kappa}{\rho C_p}\right)_{\text{water}}$, heat flows faster inside particle compared to the surrounding medium and accumulates at the boundary before it diffuses away through water. So, it can be considered that the boundary maintains a constant temperature throughout when being

continuously illuminated. Next, we estimate at what timescale the uniform temperature approximation (UTA) is valid. The typical time scale τ required to reach the steady-state regime is

$$\tau = \frac{R^2}{D} \quad (7)$$

where D is the thermal diffusivity ($m^2\ s^{-1}$) and R is typical experimental length scale. Since, the heat flow is orders of magnitude faster in metal than water, the surrounding medium governs the magnitude of transient time scale which is of the order of μs. Therefore, for a CW illumination we can consider steady-state heat equation[2]

$$\nabla \cdot [\kappa \nabla T(\mathbf{r})] = Q(\mathbf{r}) \quad (8)$$

to find out the temperature distribution in water. We assumed the thermal conductivities of Ag and water to be 429 $\mathrm{Wm^{-1}K^{-1}}$ and 0.6 $\mathrm{Wm^{-1}K^{-1}}$ respectively.

In this simulation, we neglected the effect of convection on temperature rise. We considered the Rayleigh number $Ra = g\beta\Delta T L^3/\nu\alpha$, where g is the acceleration due to gravity, β is the volume thermal expansion coefficient of water, ΔT is the simulated rise in temperature ignoring convection and therefore an upper limit for the temperature rise, L is a characteristic length of the system, ν is the kinematic viscosity and α is the thermal diffusivity of water. The value of $Ra \sim 10^{-11}$ shows role of convection in the heat flow and therefore temperature rise under present experimental conditions can be neglected.

Supplementary Note 3: Measurement of temperature rise

The temperature measurement was based on measuring the fluorescence efficiency of a water-soluble dye Rhodamine B which has a high temperature sensitivity. The fluorescence intensity $I(T)$ as a function of temperature can be written as[3]

$$\ln\frac{I(T)}{I(T_{\mathrm{ref}})} = \beta\left(\frac{1}{T} - \frac{1}{T_{\mathrm{ref}}}\right) \quad (9)$$

where T_{ref} is a reference temperature and the corresponding fluorescence intensity is $I(T_{\mathrm{ref}})$. To find the unknown constant β, we use a uniformly heated well with a uniform and constant dye concentration (10 μM solution). The calibration experiments are performed with a plate reader (Tecan-infinite M1000Pro) where the sample can be uniformly heated from ambient to 42 °C. An excitation source at 540 nm with bandwidth 5 nm was used to excite the fluorescence which showed an emission maximum at 585 nm in water. Supplementary Figure 6a, shows the variation of $\ln\frac{I(T)}{I(T_{\mathrm{ref}})}$

as a function of $\left(\frac{1}{T} - \frac{1}{T_{\text{ref}}}\right)$, which gives β = 2261 ± 103 K. This β value has been used to determine the temperatures of the ACT when illuminated at 1064 nm.

To find out the temperature of ACT we performed experiments in static conditions. First the ACT along with dye solution is dried on a glass cover slip and then water is added again on top of the ACTs stuck to the glass surface. This is to ensure the dye molecules do not diffuse in the heated region around the plasmonic disc and also to remove the background fluorescence from the bulk liquid. We have used a mercury vapor lamp with an excitation filter between 510-550 nm to excite the fluorescence. We have illuminated the plasmonic nanodisk at 1064 nm wavelength at 3 different power levels. The fluorescence intensity was measured before $\left(I(T)\right)$ and during the illumination $\left(I(T_{\text{ref}})\right)$ which shows a clear drop in the fluorescence intensity in the plasmonic (metallic) part of the ACT whereas in other areas in the field-of-view fluorescence intensity remained unchanged. For a certain laser power, the temperature measurement (solid black line) was repeated for six different ACTs and the standard deviation of such six measurements are shown as error bars in Supplementary Figure 6b. We have also performed a control experiment where the dye was illuminated by 1064 nm laser without the ACT at same power levels, and no change in fluorescence intensity with the incidence of IR-light was detected. The temperature rise in ACTs are estimated from eqn.9 and plotted in Supplementary Figure 6b (solid black line and symbols).

In the same figure we have also plotted the simulated temperature rise for both ACT and isolated nanodisk in water, corresponding to illumination wavelength of 1064 nm. The simulated temperature rise for the ACT including metallic and dielectric parts (red solid line) is slightly smaller than the isolated nanodisk (without the SiO_2 structure, dotted green line). This was due to the higher thermal conductivity of glass than water which is taken to be 1.38 $\text{Wm}^{-1}\text{K}^{-1}$ in all the calculations. The difference between simulated and experimental values can be attributed to the fact that the experimental measurements have been performed while the ACTs were immobilized on glass surface of the fluidic chamber which has higher thermal conductivity than water (0.6 $\text{Wm}^{-1}\text{K}^{-1}$).

Accordingly, we considered ACTs located at the interface between two media, where we assumed an average thermal conductivity, $\kappa_{\text{avg}} = \frac{\kappa_{\text{water}} + \kappa_{\text{glass}}}{2}$. We assume $\kappa_{\text{avg}} = 1\ \text{Wm}^{-1}\text{K}^{-1}$ in eqn.8 then the calculated temperature rise (dashed blue line) is close to and slightly less than the experimentally measured values. The overall agreement between the experimental measurements and the numerical models is encouraging.

We must stress the lower temperature rise in our experiments is to a large extent due to use of Ag as the plasmonic material, as opposed to Au commonly used for thermoplasmonic experiments. For example, higher temperature rise (~33 K at 10 $\text{mW}\mu\text{m}^{-2}$) was found in literature[4] for similar disk geometry (288 nm dimeter, 40 nm thickness) fabricated on glass substrate and immersed in water but

where the discs were made with gold (which is a better absorber than Ag). Also, in a separate plasmonic trapping experiment 37 K rise in temperature above ambient was reported for Au disk nanoantenna[5]. The heating of a silver nanoparticle has been compared with gold in a previous literature which also predicted lower temperature rise for Ag (see Supplementary information of Ref# 6) at IR wavelengths[6].

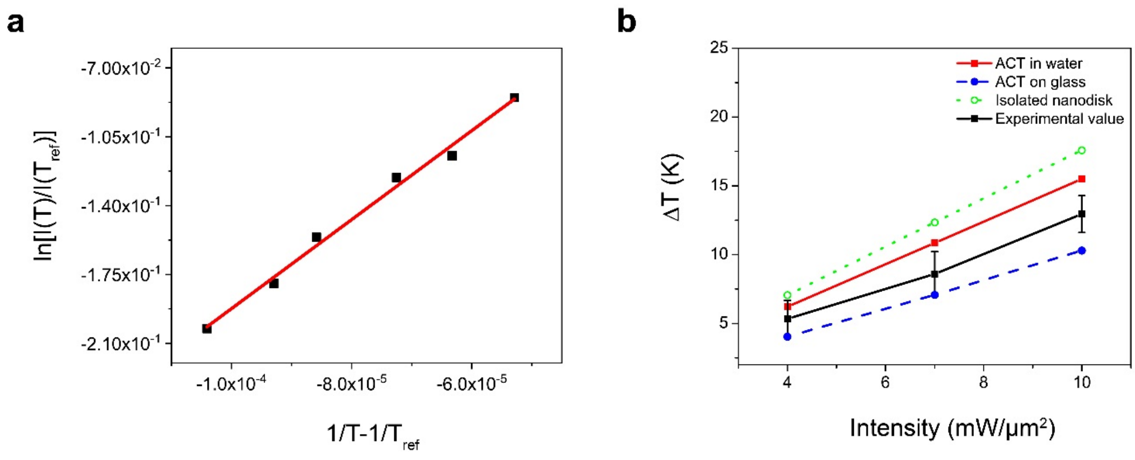

Supplementary Figure 6: Measurement of temperature rise (a) Fitting of the temperature calibration data of Rhodamine-B. The slope gives the value of β. (b) Simulated and experimental measure of temperature for three different power levels. The different simulation conditions are discussed in the adjoining text.

Supplementary References:

1. Johnson, P. B. & Christy, R. W. Optical Constants of the Noble Metals. *Phys. Rev. B* **6**, 4370–4379 (1972).

2. Baffou, G., Quidant, R. & García de Abajo, F. J. Nanoscale Control of Optical Heating in Complex Plasmonic Systems. *ACS Nano* **4**, 709–716 (2010).

3. Lemoine, F., Antoine, Y., Wolff, M. & Lebouche, M. Simultaneous temperature and 2D velocity measurements in a turbulent heated jet using combined laser-induced fluorescence and LDA. *Exp. Fluids* **26**, 315–323 (1999).

4. Baffou, G. *et al.* Thermal Imaging of Nanostructures by Quantitative Optical Phase Analysis. *ACS Nano* **6**, 2452–2458 (2012).

5. Ndukaife, J. C. *et al.* Long-range and rapid transport of individual nano-objects by a hybrid electrothermoplasmonic nanotweezer. *Nat. Nanotechnol.* **11**, 53–59 (2016).

6. Melzer, J. E. & McLeod, E. Fundamental Limits of Optical Tweezer Nanoparticle Manipulation Speeds. *ACS Nano* **12**, 2440–2447 (2018).
