# Supplementary material for: All optical dynamic nanomanipulation with active colloidal tweezers
Source: Nat Commun. 2019 Sep 13;10:4191. doi: 10.1038/s41467-019-12217-2 (PMC6744401; doi:10.1038/s41467-019-12217-2)
Supplement: Supplementary file 3 — Description of Additional Supplementary Files [file 41467_2019_12217_MOESM3_ESM.pdf]

Description of Additional Supplementary Files

File name: Supplementary Movie 1
Description: Trapping of 100 nm fluorescent nanodiamonds by an ACT.

File name: Supplementary Movie 2
Description: Demonstration of trap, transport and release of a 200 nm fluorescent polystyrene particle in ionic solution. Video played at 2X of original speed.

File name: Supplementary Movie 3
Description: Demonstration of trapping of 400 nm polystyrene particle in vertical configuration.

File name: Supplementary Movie 4
Description: Demonstration of trapping of 400 nm polystyrene particle in horizontal configuration.

File name: Supplementary Movie 5
Description: Collective manipulation of 400 nm polystyrene particle using multiple ACTs using a defocused optical tweezer. Video played at 0.5X of original speed.

File name: Supplementary Movie 6
Description: Demonstration of trapping of 40 nm fluorescent polystyrene particle in vertical configuration.

File name: Supplementary Movie 7
Description: Demonstration of trapping of 40 nm fluorescent polystyrene particle by an ACT attached to substrate (in horizontal configuration).

File name: Supplementary Movie 8
Description: Demonstration of parallel and independent control in manipulation using 300 nm fluorescent magnetic particle.
